# Supplementary figures and images for: Structure of scavenger receptor SCARF1 and its interaction with lipoproteins
Source: eLife. 2024 Nov 14;13:RP93428. doi: 10.7554/eLife.93428 (PMC11563577; doi:10.7554/eLife.93428)

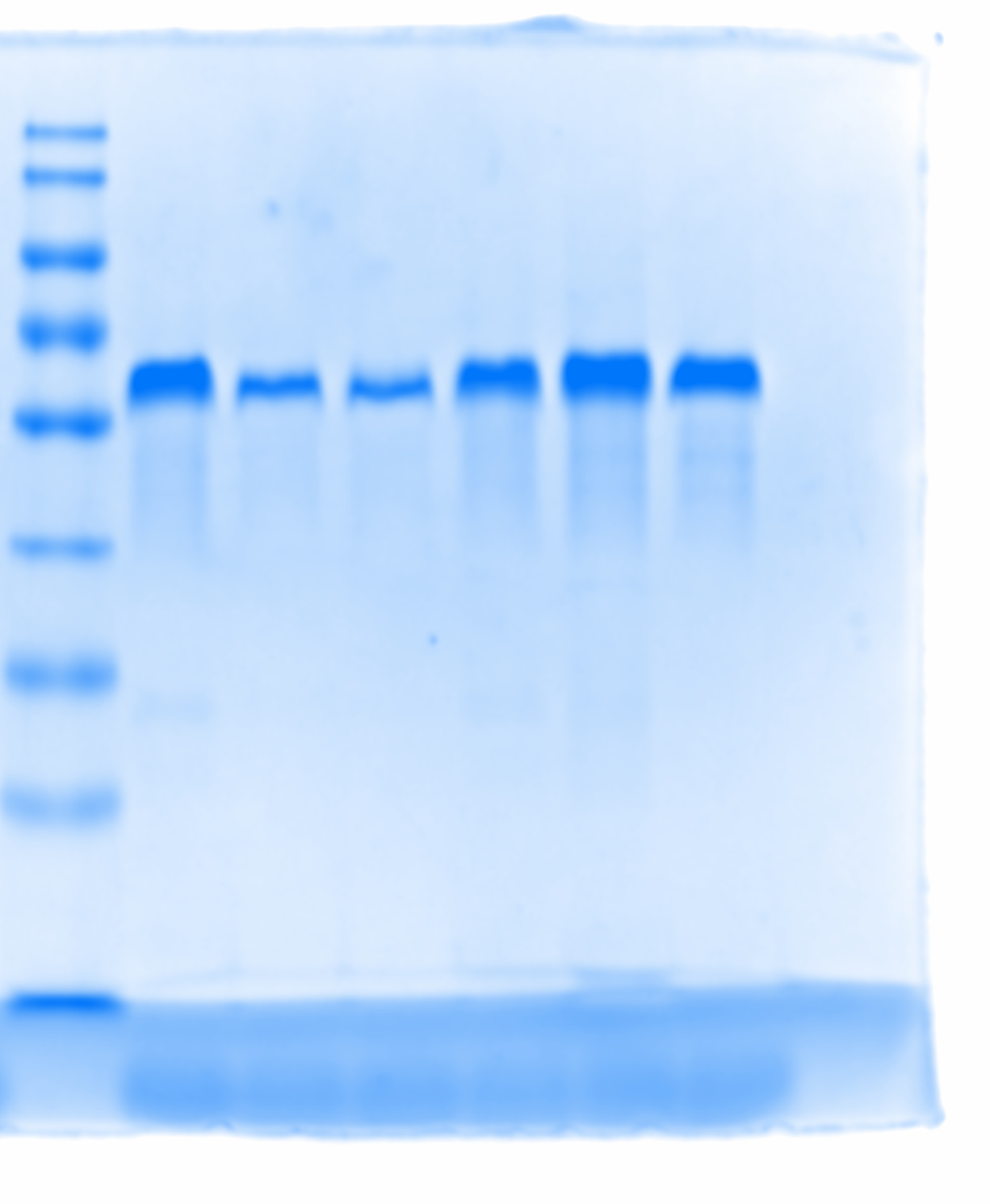

Supplement: Figure 2—figure supplement 2—source data 1. [file elife-93428-fig2-figsupp2-data1.zip › Figure 2-figure supplement 2-source data 1/AKTA-fengtu-2.tif]
