# Supplementary material for: Structure of scavenger receptor SCARF1 and its interaction with lipoproteins
Source: eLife. 2024 Nov 14;13:RP93428. doi: 10.7554/eLife.93428 (PMC11563577; doi:10.7554/eLife.93428)
Supplement: Figure 2—figure supplement 2—source data 2. [file elife-93428-fig2-figsupp2-data2.zip › Figure 2-figure supplement 2-source data 2/Figure 2-figure supplement 2-source data 2.pdf]

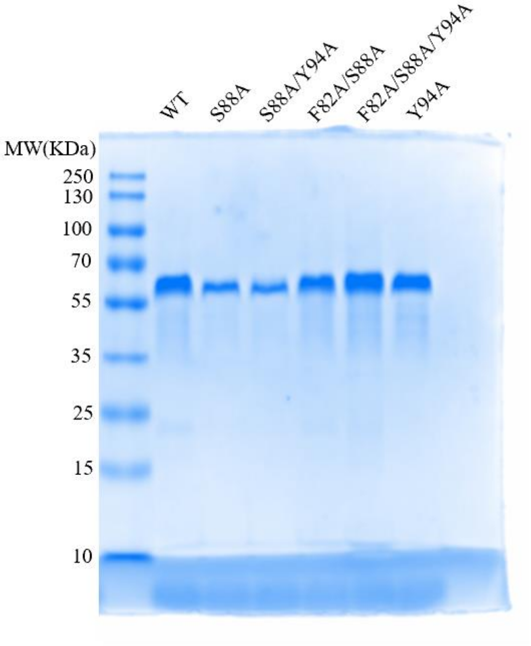

Figure 2-figure supplement 2-source data 2. Original gel corresponding to Figure 2-figure supplement 2, panel A. Lane 4 (F82A/S88A) is not shown in Figure 2-figure supplement 2.
