# Supplementary material for: Structure of scavenger receptor SCARF1 and its interaction with lipoproteins
Source: eLife. 2024 Nov 14;13:RP93428. doi: 10.7554/eLife.93428 (PMC11563577; doi:10.7554/eLife.93428)
Supplement: Supplementary file 1. [file elife-93428-supp1.docx]

| Supplementary File 1. X-ray data collection and processing | | |
| --- | --- | --- |
| Protein | 20-132aa of SCARF1 | 20-221aa of SCARF1 |
| Beamline | SSRF BL18U1 | SSRF BL18U1 |
| Wavelength (Å) | 0.98 | 0.98 |
| Space group | P2_1_2_1_2_1_ | P4_1_22 |
| Cell parameters |  |  |
| a, b, c (Å) | 47.01, 50.70, 110.74 | 102.01, 102.01, 83.61 |
| α, β, γ (°) | 90, 90, 90 | 90, 90, 90 |
| Resolution (Å) | 30.00-2.10  (2.18-2.10) | 30.00-2.60  (2.69-2.60) |
| R_merge_ | 0.075(0.398) | 0.088(0.795) |
| R_pim_ | 0.023(0.149) | 0.018(0.176) |
| Unique reflections | 16105(1567) | 14015(1299) |
| I/σ (I) | 33(3.03) | 45.5(2.75) |
| Completeness (%) | 99.9(94.6) | 99.2(93.4) |
| Multiplicity | 9.9(7.5) | 23.3(17.9) |

Values in parentheses are for the highest-resolution shells.
