# Supplementary material for: Structure of scavenger receptor SCARF1 and its interaction with lipoproteins
Source: eLife. 2024 Nov 14;13:RP93428. doi: 10.7554/eLife.93428 (PMC11563577; doi:10.7554/eLife.93428)
Supplement: Supplementary file 2. [file elife-93428-supp2.docx]

| Supplementary File 2. Crystallographic statistics of the structures | | |
| --- | --- | --- |
| Protein | 20-132aa of SCARF1 | 20-221aa of SCARF1 |
| Resolution (Å) | 24.71-2.20(2.28-2.20) | 26.89-2.60(2.69-2.60) |
| R_work_ | 0.219(0.244) | 0.229(0.339) |
| R_free_ | 0.252(0.292) | 0.246(0.347) |
| Protein atoms | 3223 | 1133 |
| Wilson B-factor (Å^2^) | 38.8 | 71.2 |
| Average B, all atoms (Å^2^) | 56.0 | 86.0 |
| Rmsd bonds (Å) | 0.36 | 0.43 |
| Rmsd angles (°) | 0.60 | 0.71 |
| Ramachandran favored (%) | 95.41 | 95.36 |
| Ramachandran outliers (%) | 0 | 0 |
| PDB code | 8HN0 | 8HNA |

Values in parentheses are for the highest-resolution shells.
